# Environmental Impact of Nanoparticles’ Application as an Emerging Technology: A Review

**DOI:** 10.3390/ma14010166

**Published:** 2020-12-31

**Authors:** Guillermo Martínez, Manuel Merinero, María Pérez-Aranda, Eva María Pérez-Soriano, Tamara Ortiz, Belén Begines, Ana Alcudia

**Affiliations:** 1 Department of Organic and Medicinal Chemistry, Faculty of Pharmacy, University of Seville, C/Profesor García González, 2, 41012 Seville, Spain; mtnezmun@gmail.com (G.M.); lolo191995@gmail.com (M.M.); mariapar89@gmail.com (P.-A.M.); 2 Department of Materials Science and Engineering and Transport, Escuela Politécnica Superior, University of Seville, 41011 Seville, Spain; evamps@us.es; 3 Department of Normal and Pathological Cytology and Histology, Faculty of Medicine, University of Seville, 41009 Seville, Spain; tortiz@us.es

**Keywords:** nanotechnology, nanoparticles’ emission, nanomaterial release models, negative impact, positive impact, toxicity, green chemistry

## Abstract

The unique properties that nanoparticles exhibit, due to their small size, are the principal reason for their numerous applications, but at the same time, this might be a massive menace to the environment. The number of studies that assess the possible ecotoxicity of nanomaterials has been increasing over the last decade to determine if, despite the positive aspects, they should be considered a potential health risk. To evaluate their potential toxicity, models are used in all types of organisms, from unicellular bacteria to complex animal species. In order to better understand the environmental consequences of nanotechnology, this literature review aims to describe and classify nanoparticles, evaluating their life cycle, their environmental releasing capacity and the type of impact, particularly on living beings, highlighting the need to develop more severe and detailed legislation. Due to their diversity, nanoparticles will be discussed in generic terms focusing on the impact of a great variety of them, highlighting the most interesting ones for the industry.

## 1. Introduction

Human beings have been using nanoparticles (NPs) since ancient history. Researchers have reported examples of NPs uses from 4500 years ago, such as the application of nanofibers in ceramics or the production of “Egyptian blue”, the most ancient synthetic pigment known, obtained from the mixture of quartz and nanoparticulate glass [1]. In 1857, Michael Faraday did the first modern scientific description of NPs by observing the difference between optical properties of colloidal gold and ordinary metal [2]. Nevertheless, it was not until 1959 that Richard P. Feynman, in an American Physical Society meeting, introduced the concept of what later will be called “nanotechnology” by mentioning nanoscale materials manipulation [3]. In 1974, N. Taniguchi introduced this term for the first time in order to describe the processes and mechanisms involved in creating nanoscale materials [4]. From this point on, developments in this field have been extending and applying nanotechnology concepts to a wide spectrum of researching fields. Since the 80s, nanotechnology’s “golden-age” started with the discovery of fullerene and was supported by the publication of Eric Drexle’s book “Engines of Creation: The Coming Era of Nano-technology”. The interest in this science and its applications in different fields went up further at the beginning of the 21st century, becoming a major priority issue in the United States of America [3]. In this sense, the USA approved the “National Nanotechnology Initiative” (NNI) in 2001, with the aim of boosting local nanotechnology development. Since then, many other countries such as Japan or European countries have created their own departments in order to impulse nanotechnologies and their possible developments [5].

**Figure 1:**
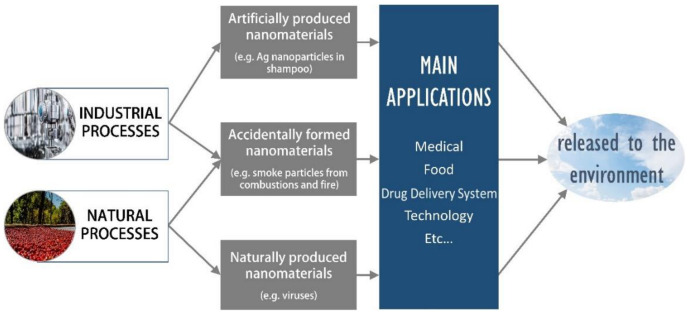
Nanoparticles classification and main applications.

Nowadays, nanotechnology is gaining a lot of interest from industry thanks to the emergence of new applications in different fields such as medicine, electronics, or agriculture. This increasing interest is leading to a bigger production of NPs in the industry and places us in uncommon new scenarios where the impact they might have on the environment and living beings needs to be urgently assessed. To clearly understand the potential benefits or risks NPs may cause to the environment, some concepts need to be clarified. Nanoscience is defined as the field of science that studies nanometric scale matter, considering its size and characteristics. However, nanotechnology investigates ways of manipulating and controlling these properties [5]. The term NPs refers to particles whose sizes are in the nanometre scale for some authors and for others it includes the shortest definition of nanoparticles, which is probably the most intuitive one, taking into consideration only their size, which is limited conventionally to about 100 nm in any direction [6]. This small size generates a wide range of applications in many different research fields [7]. Properties presented in this size are unique and differ from those present in higher sizes. For instance, depending on NPs size, gold is able to raise its melting point from 200 to 1068 °C [7]. Although different NPs’ classification methods may be found in the literature attending to different characteristics [8], it is important to differentiate nanoparticulate materials according to their origin for the purpose of this review. In this sense, three different groups can be established (Figure 1) [5]:

- Accidentally formed nanomaterials: They appear as a subsequent product from industrial or natural processes, such as combustions (e.g., smokes from cigarettes or fires).
- Artificially produced nanomaterials: They are designed by humans with determined properties and characteristics (e.g., Ag NPs in shampoos) [9]. The main difference between accidentally formed nanomaterials is that these ones are intended to be formed with chosen sizes and composition, like their characteristics, while accidentally formed nanomaterials appear in a natural and spontaneous way.
- Naturally produced nanomaterials: They can be found in living beings and nature (e.g., viruses). The line that separates natural or accidental materials is, in certain occasions, extremely difficult to distinguish [10].

The number of investigations released and published as well as their utilities and applications have risen gradually in the last two years (Web of Science recorded 1581 new reports about NPs in 1999 versus the 81,303 recorded in 2019). For this reason, to assess the impact NPs may have in the environment, it is essential to know their recent applications, mainly those with a higher value for the actual society (Figure 1):

- Antibacterial activity: The threatening increase of microorganisms resistance towards antibiotics has become an important concern, where metallic NPs can make the difference as an alternative or adjuvant treatment due to their antibacterial properties [11]. For example, zinc oxide inhibits and prevents *Staphylococcus aureus* growth. Unfortunately, this mechanism is still not fully understood [12], although ZnNPs has been described to cause growth inhibition in bacteria via reactive oxygen species (ROS) production, specifically H_2_O_2_ [13]. In *E. coli,* these nanoparticles are accumulated in the membrane [14], generating electrostatic charges that cause critical damage [15].
- Drug delivery systems: Delivering a drug to a specific site where it is meant to exert its effect is one of the greatest nanotechnology promises [16], becoming especially important when it is used for cancer treatment in order to avoid associated side effects and problems. Interestingly, there are some approved therapies based on the use of these technologies, such as albumin NPs [17], for example, Abraxane^®^ [18]. In particular, the enhanced permeability and retention effect (EPR effect) plays an important role in the biological distribution of NPs, granting high drug concentration in tumor cells against very low drug concentration in healthy tissues, which results in a higher therapeutic effect and desirable toxicity [19]. The possibility of transporting different substances at once granted by nanomaterials is a stimulating advantage and a potential solution for multiple therapies [20].
- Food preservation: Some NPs’ properties allow them to form an impassable barrier against gases, humidity and other factors that could alter and reduce food stability. Furthermore, the food decomposition preventing effect could be complemented due to the antibacterial and antioxidant activities that some NPs possess [21].
- Sunscreen: Titanium dioxide (TiO_2_) and zinc dioxide (ZnO) in a nanometric scale are extremely effective at absorbing ultraviolet light [22], which makes them a valuable component for sunscreens and solar protection products.
- Molecular detection and diagnosis: The use of magnetic NPs for detecting specific molecules inside the organism has accomplished a lot of achievements, making it possible to identify whether pathogens are invading the organism [23] or molecules related to inborn genetic defects [24,25]. For instance, gold NPs (Au NPs) are used in different biological analysis processes, such as diagnosing patients with possible allergy problems (InmunoCAP^®^, ImmunoCap™ Phadiatop^®^, Phadia AB, Upssala, Sweden) or as highly visible indicators in pregnancy tests (First Response^®^, Church and Dwight Co. Inc., Princeton, NJ, USA) due to their optical properties and their chemical stability [26]. Gadolinium NPs (Gd NPs) are also widely used in cancer diagnosis [27].
- Sports equipment: carbon nanotubes allow enhancing equipment and tools by improving resistance and flexibility, granting and enduring a more effective product [28].
- Renewable energy sources: some studies have demonstrated that nanofluids [29] and photovoltaic devices with nanocrystals [28] can increase solar energy harvesting.
- Water purification: iron NPs slightly enriched with Palladium have the capacity to eliminate organic chlorine in waters and soils [30].
- Agriculture: among some uses of NPs in agriculture, some examples can be highlighted. Thus, in biofortification, iron oxide NPs are applied [31]; or their use as pesticides, being especially interesting the employment of Cu(OH)_2_ NPs as a respectful pesticide with the environment [32].
- Other applications: bone reconstruction [33], car tires reinforcements, speakers heat transfer [34], etc.

The unique properties and characteristics of NPs that grant their special effects are also associated with living beings’ health and environmental risks that must be considered in order to make responsible and balanced use of them. It is clearly observable that, in the case of TiO_2_, its small size predisposes its surface to be photoreactive, causing microorganisms oxidation as well as unwanted oxidative damage to other organisms like fishes [35]. Despite the existing investigations aimed to clarify, classify and prevent these risks, estimation of long-term consequences with high quality is not yet achieved and, more efforts will be required in order to reach these necessary goals [28]. According to NNI, the budget assigned by the US government in 2018 was $1,740.9 million, of which only $10.6 million were designated to environmental protection agency, corresponding to 0.6% of the total budget [36]. On the other hand, the heterogeneity that these compounds present is an added complication when it comes to evaluating them as a whole set. In addition, calculating their environmental impact involves numerous parameters from disparate fields, requiring a group of experts from different scientific areas; physicists who study their structure, environmental engineers who quantify the amount of material released to the environment, chemists, biologists, toxicologists who evaluate their side or toxic effects, etc. [37]. In consequence, this current NP boom and the limited efforts and investments concerning their possible environmental consequences make it necessary to focus on this specific aspect of nanotechnology, because the environmental damage can have direct consequences in human’s health and that NPs can easily enter in the human body [38].

Therefore, this review intends to give an overview of the potential transformations that NPs can undergo in their life cycle and their environmental impact, focusing on the different mechanisms of toxicity they may produce on living beings. However, the environmental impact of the use of nanoparticulate systems is not always negative and some interesting effects will be discussed as well as an analysis of the necessity of specific changes in the current legislation to reduce their negative environmental footprint.

## 2. Nanoparticles Emission

**Figure 2:**
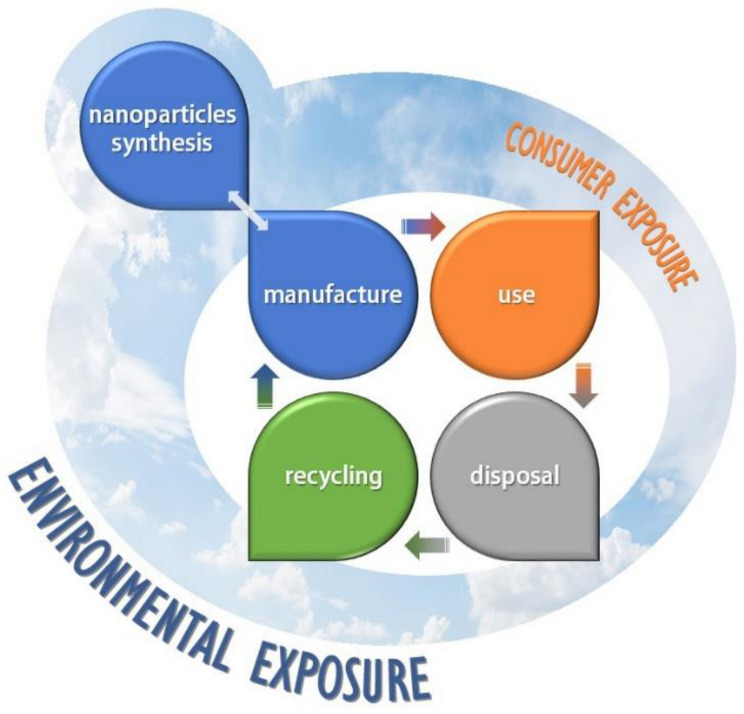
The life cycle of nanoparticle-containing products.

Understanding the NP environmental impact requires detailed comprehension of the characteristics of these systems such as identification, physicochemical properties, including how they are emitted to the environment and their toxicity in living beings [39]. The inherent potential toxicity of the NPs is fundamentally conditioned by their ability to reach and invade the different environmental compartments (earth, water, air). This impact is directly related to the number of NPs released to the biosphere. Therefore, the risks associated to the use of nanomaterials are determined by all those processes that control their release into the environment, the shipment between installations and zones or between organisms, due to the food chain, and the transformations that can occur once they are released [40]. Evaluating and quantifying the amount of released nanomaterial requires exhaustive research of its entire life cycle [41], starting from the nanomaterials production processes and ending with the recycling and disposal procedures, taking into account how they are incorporated into the final products and how they are utilized (Figure 2) [42].

Although nanomaterials release could happen in any stage of the life cycle, it becomes a major issue when NPs are employed in the final product (e.g., fabric or clothes washing or NPs-containing aerosol employment). This fact makes the impact evaluation an even harder task, as it is the most difficult phase of the life cycle [38] due to the impossibility of establishing protocols as well as the existence of many different uncontrolled variables, such as inadequate employment, different climates, etc., [43].

### 2.1. Product Fabrication

From the first production stages, emissions of NPs can easily occur, especially when variables and procedures are not strictly controlled. Two different types of accidental emissions may happen: direct emissions (e.g., open windows when working with powder materials or an accidental spill) and indirect emissions (e.g., incorrect waste treatment). During the manufacturing stage of the final products, the emission increases when processes of structural modification of the material containing NPs take place, such as cuts, drilling or procedures that require high energy and temperature to fabricate adjusting the shape [44]. Exposure of workers to NPs during the handling and production is a possible route of emission into the environment, although its relevance still remains unknown [45].

### 2.2. Product Employment

NPs emissions during product employment can happen intentionally or accidentally. While the origin or source and the number of NPs released to the environment in intentional emissions are known and measurable, these parameters can only be estimated when it comes to accidental release caused by deterioration and alteration of products. Depending on the type of product and its properties, the emission will be different [46]. Thus, from fluid products, almost the entirety of the NPs is quickly released when the product is employed [47]. However, from solid products, the contained NPs are gradually released during the product employment (e.g., NPs used in pneumatic tires are released with attrition and friction) [48]. From spray products, the total emission of NPs is immediate [49], while from suspensions, the total emission occurs in the first hours (e.g., cosmetics or sunscreens). Finally, NPs can remain stable for years in clothes and paint dyes.

### 2.3. Disposal and Recycling

The way waste treatment is carried out affects the amount and the way in which NPs are released from the products. These products can be dumped, incinerated, or recycled.

- Dumped products: The landfill is the place where, usually, most of the nanomaterials end [41]. Depending on the resistance of the products to degradation and the landfill control systems, they may or may not release the nanomaterials to the environment [50].
- Incinerated products: Their incineration causes the appearance of ash particles in the air, and NPs could form an important part of their composition [41]. However, the efficiency of the filters present in incineration plants (higher than 99.6%) causes an extremely small direct emission of these particles to the air [51].
- Recycled products: The emission can occur when recycling processes are carried out depending on their resistance [50]. For example, TiO_2_ remains stable when acids are used in a recycling process, but ZnO dissolves completely and carbon nanotubes can be degraded and destroyed [43].

### 2.4. Transformation

In recent years, several studies about the transformation processes of nanomaterials have been published highlighting the idea that, while quantification of the released NPs may be representative of the manufacturing stage, modified nanoparticulate materials are almost exclusively the ones that will reach the environment. For this reason, it is necessary to study the transformations that an NP can suffer in order to have a real vision of what is really emitted to the biosphere [52]. These transformations directly affect the toxicity and impact that released nanomaterials may cause. For example, when silver NPs (Ag NPs) are in solution, Ag is oxidized (Ag^0^ turns into Ag^+^), producing antimicrobial effects [53]. Transformations can happen in any life cycle phase, from production and storage stages. For example, unprotected ZnO powders can double its size after four years exposed to air [54]. On the other hand, transformations that nanomaterials can experience have several and different reasons and mechanisms, producing NPs that differ from the original ones in physical or chemical parameters [55]:

A. Photochemical transformation: depending on the incident wavelength, the penetration capacity on the product and the nanomaterial photosensitivity, the excitation produced in the NPs and their consequent transformation can be greater or lower. For example, the interaction of TiO_2_ NPs with sunlight produces the appearance of ROS in living organisms, causing an increase in toxicity [37].
B. Oxidation and reduction: these processes can occur when the reaction is thermodynamically favored, so they depend on the medium and conditions surrounding the nanomaterial (pH, presence of oxidizing and/or reducing agents, reagents, or stabilizers). For example, the oxidation of Ag^0^ to Ag^+^ is a consequence of washing clothes and fabrics that include NPs made of this material. This fact increases their toxicity, in addition to reducing their effectiveness in the product [56].
C. Dissolution and precipitation: the dissolution of ions or water-soluble molecules can occur, but also subsequent precipitation of a new solid which contains, in addition to NPs, other ligands naturally present in the water. Therefore, dissolution and precipitation processes are transformations that may happen independently or consecutively. For example, the dissolution of metallic NPs made of Cu or Zn increases their bioavailability and, therefore, their toxicity [57].
D. Adsorption and desorption: adsorption to solids can take place through Van der Waals forces, electrostatic interactions, or chemical bonds, while changes in the balance between products cause desorption. For example, graphene oxide (GO) NPs may adsorb antibiotics such as levofloxacin, manipulating their mobility, transport and effect, increasing their risk of toxicity [37].
E. Combustion: high temperatures processes, such as incineration, lead to combustion reactions that can chemically modify the NPs. For example, iron NPs in spontaneous coal combustion affects climatic variables [58].
F. Biotransformation: it includes all the processes listed above (except combustion) when they are mediated by biological agents. For example, myeloperoxidase in humans degrades graphene oxide NPs, reducing their cytotoxicity [37].
G. Abrasion: Common handling of some products can produce the release of NPs contained in their structure. For example, building materials coatings or polyurethane coatings can release NPs after long-term employment [55].

**Figure 3:**
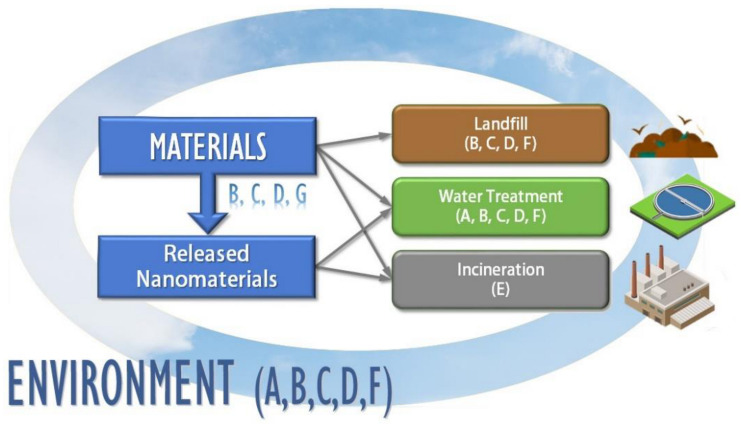
Life-cycle of Ag nanoparticles in socks and their transformations in the different stages.

In order to expose and illustrate these transformation processes, Nowack et al. [55] carried out an interesting study that exemplifies all the transformation processes that can occur in the life cycle of a product that contains nanomaterials, in this case, Ag in socks. Ag-derived NP materials are used in numerous products to reduce smells and the proliferation of infectious agents, such as bacteria. Several studies have shown that the Ag NPs in these products may be released in significant amounts when washed, so they will be transported with the used water. If the water treatment is incorrect, these NPs can end up in the sea where, depending on their concentrations, they present a risk to the health of aquatic organisms. Many NPs transformations also take place in these washing processes due to the use of detergents, agitation and temperature changes. As displayed in Figure 3, the main processes that affect Ag NPs are those that take place during tissue washing: oxidation (B), dissolution and precipitation (C), adsorption and desorption (D) and abrasion (G). Photochemical transformation (A) and biological transformation (F) processes are also possible in water treatment. Finally, when waste is incinerated, the release of transformed NPs by combustion (E) can happen. Once released and transformed, the toxicity of these new compounds seems to be related to physicochemical parameters, in addition to the concentration and elements of the environment, such as pH or the presence of ligands [55].

In order to get a more realistic image of the way in which NPs are released and deposited in the environment, it is necessary to perform additional studies on the transformations that nanomaterials undergo during their life cycle, since these changes can increase the toxicity they may cause. The development of functional and validated evaluation procedures will clarify the potential consequences that nanotechnology can have on living beings and the environment [43].

### 2.5. Evaluating Nanomaterial Release

**Figure 4:**
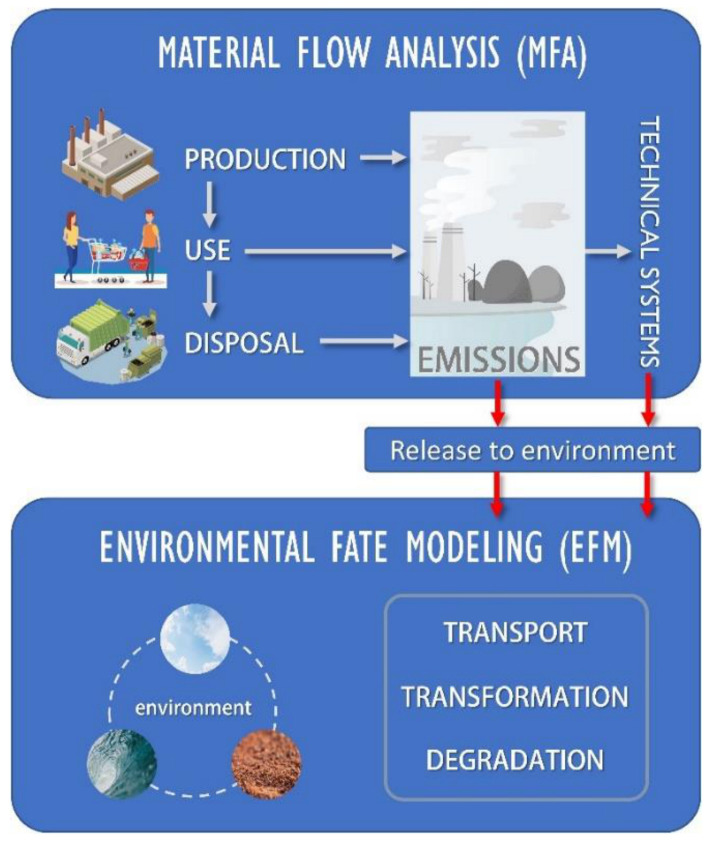
Schematic of how Environmental Fate Models (EFM) models are related to Material Flow Analysis (MFA) models.

In order to evaluate the emission of NPs into the environment, different models and approaches have been used, whose methodologies vary substantially both in conceptualization and in data collection; from more general models to models that are used for a specific scenario of a determined nanomaterial [46]. Most commonly used models are Material Flow Analysis (MFA) [40], which are used to predict the product emissions to technical compartments (landfills, recycling plants, water treatments plants, etc.) and, from there, to the environment; and the Environmental Fate Models (EFM) [59], which describe the transport and distribution through the different environmental compartments (water, earth, air). In other words, MFA focuses on emissions produced during the life cycle of the product and where they are finally deposited, while EFM describes how NPs, once emission has taken place, are transported, transformed and degraded between the different environmental compartments [41] (Figure 4).

The application of these models is conditioned and determined by the specific compartments studied for each singular case, as well as the way to collect the data. Usually, the information obtained is not totally accurate, and probabilistic statistical methods have to be employed in order to achieve a higher reality fidelity [41]. Despite MFA being the first models used to describe the emission of NPs to the environment, they are not the most appropriate to evaluate the concentrations of NPs released. MFA models deal with simplified assumptions where parameters such as size, shape, or phase shifts are not considered, making EFM models more suitable in this situation.

The different EFM models can be categorized according to whether they are constant or variable models over time, or whether the transformations of the NPs are dependent on their properties, environmental conditions, or both. The way an EFM model is proposed can drastically affect the results [59]. Some of the most remarkable models are SB4N model and RedNano model, both of which include the evaluation of transformation processes due to solution; and the Stream-Flow model, which specifically contemplate potential chemical transformations of Ag and ZnO NPs [41].

In spite of the recent advances and developments that researchers have achieved and a decrease in the limitations related to these studies, the predictive ability of these models is still limited, therefore any result obtained by the use of these methods has to be considered as a probability. Nevertheless, if studies and investigations in this field allow understanding NPs (their life cycle and the processes and transformations that suffer when they reach the environment) these models could be used as a good basis for decision-making to reduce emissions in a near future [59].

## 3. Environmental Negative Impact

Once they reach the environment, the impact of NPs is directly related to their ability to accumulate in organisms, harming them [60]. Organisms are naturally exposed to nanomaterials, but it does not mean they are innocuous; indeed, they can have a very harmful effect depending on the circumstances. Besides, while naturally originated NPs are usually joined over time and form higher size materials, manufactured NPs tend to persist due to the use of surfactants and stabilizers [61,62]. Because of that, an evaluation of how the employment of these materials could affect the environment is needed [63].

### 3.1. Absorption and Distribution

NP absorption by microorganisms takes place through the cell surface, but in living beings with a higher level of complexity, it usually takes place through the respiratory system, the gastrointestinal system or the skin. The way NPs enter the organism depends directly on the characteristics of living beings. For example, cylinder-shaped carbon nanotubes can enter easily the human body through the pulmonary epithelium and cause toxicity, while other carbon NPs, like fullerenes, do not cause toxicity [64]. The prokaryotes cell surface provides some protection to organisms against many types of NPs since they have no mechanisms for transferring particles through the cell wall. However, the eukaryote cell surface is able to absorb many different particles from the surroundings through endocytosis or phagocytosis, making it easier for the potential toxic NPs to penetrate and exert their harmful effects [65]. For instance, Wang et al. [66] described several parameters that determine how NPs are absorbed in seaweed cells, especially highlighting the importance of the NP size for entering through the pores of the cell wall (5–20 nm), the components that may help in absorption (glycoproteins and polysaccharides) and the membrane modifications due to cellular stage. Furthermore, Wang et al. also described how the environment and its changes (such as pH variations) affect this absorption process.

**Figure 5:**
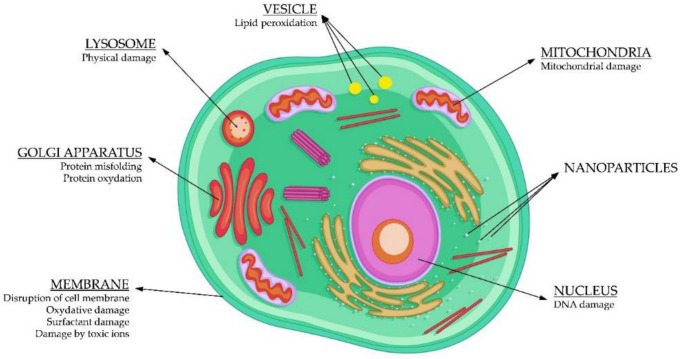
Toxic effects on the different intracellular objectives.

Once inside the cell, the toxicity and damage caused to different cell compartments (Figure 5) will depend on the type of NPs. For example, carbon nanotubes usually produce toxicity in mitochondria [67], while Ag NPs generally bind to the membrane, modifying its permeability and morphology [68]. Lysosomes are organelles where NPs end to be excreted out from the cell, but this congregation can be a source of toxicity too. Nuclei damage occurs when NPs are able to diffuse through the nuclear membrane and generate disturbances in the normal function of the Golgi complex [69]. Most of the toxic effects produced by NPs involve the production of ROS (Figure 5).

### 3.2. Mechanisms of Toxicity: Oxidative Stress

**Figure 6:**
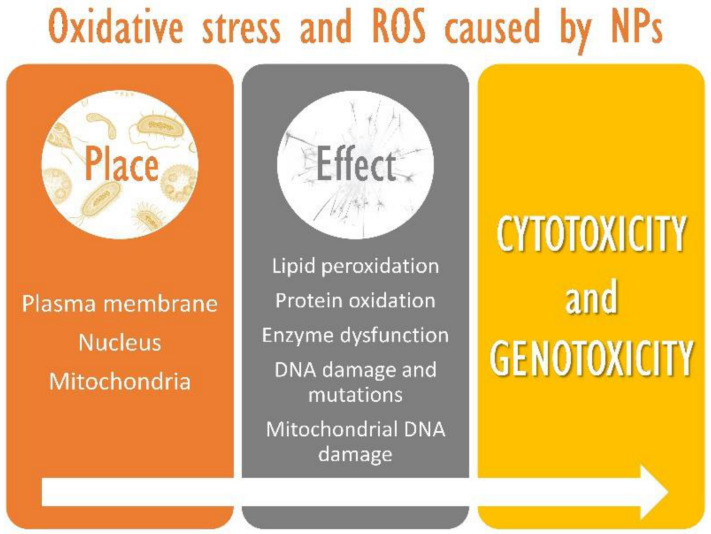
Potential cellular toxic effects of nanoparticles (NPs) caused by reactive oxygen species (ROS).

The formation of ROS, and the consequent oxidative stress, is the NPs’ principal mechanism of toxicity, even if exposure doses are low [70,71]. ROS are produced in oxidant organelles, such as mitochondria, and in acidic conditions, such as in the lysosome inner medium. NPs easily interact due to their high surface and reactivity. Media containing superoxide anions (O^2−^), hydroxyl radicals (**·**OH) and hydrogen peroxide (H_2_O_2_) produce ROS that reacts with the proteins, molecules and compartments inside the cell causing damage in different ways depending on the amount generated and the cellular management of this oxidative stress [72] (Figure 6).

ROS production caused by NPs can also be induced by interaction with cells of the immune system, such as alveolar macrophages or phagocytic cells, which can produce ROS using the NADPH oxidase enzyme complex [73]. In addition, its excess is responsible for alterations in proteins, lipids and nucleic acids that can lead to cell death. Some studies with silica NPs have demonstrated that the formation of ROS and induced cytotoxicity due to lipid peroxidation of the cellular membrane [74]. The assays evidenced dose-dependent cell membrane damage. Ahktar et al. demonstrated that a 50 μg/mL concentration of silica NPs did not produce significant damage. However, when the concentration increased to 100, 200 and 400 μg/mL, the damage rose considerably [75]. In this way, the increase of ROS is capable of modifying DNA, leading to chain disruption, DNA adducts and the formation of bridges between chains that, when not repaired, cause damage, mutations and death cell [72,76]. Additionally, several investigations conclude that oxidative stress generated by silver nanomaterials induce several DNA mutations and apoptosis cell, generated by the interaction of ROS with mitochondrial membrane phospholipids and alteration in membrane potential [73,76].

### 3.3. Toxicity in Bacteria Studies

As well as having essential functions in different ecosystems, bacteria are part of the basic food chain. Their importance, together with their easy handling, make them an essential starting point for the studies on the potential NPs’ environmental toxicity [53]. Interestingly, there are serious differences in the bacterial susceptibility to diverse nanomaterials. The different structure and composition of the cell walls in Gram-positive and Gram-negative is a key factor. Although the toxicity mechanisms in bacteria are poorly known, it has been proven that NPs clearly affect the cell walls and that oxidative stress, metal ion release and non-oxidative mechanisms are certainly involved [77]. Most of the investigations related to toxicity are based in the growth and development of bacterial communities in different environments (A–D), although there are some other studies performed with higher exhaustiveness in toxicity mechanisms that entails specific bacteria strains (E–G). In this sense, Ge et al. [78] evaluated the effect, in different doses, of TiO_2_ NPs (0, 0.5, 1.0, and 2.0 mg/g soil) and ZnO NPs (0.05, 0.1 and 0.5 mg/g soil), in incubated soil samples, by pyrosequencing. The effect produced in bacteria was demonstrated to be dose-dependent. The rate of growth of communities such as *Rhizobiales* or *Methylobacteriaceae* decreased due to the toxicity of the NPs, while, in some other samples, such as *Streptomyces*, these nanomaterials induced a higher development and an increased rate of growth, probably as a consequence of a decrease in competitive organisms, which leads to higher availability of nutrients. These studies proposed a double toxicity mechanism: direct toxicity due to cellular damage and indirect toxicity due to alterations in nutrients and soil properties. Likewise, Singh et al. [79] exposed *Deinococcus radiodurans* to ZnO NPs in concentrations between 1 and 80 μg/mL. Genotoxicity and cytotoxicity assays demonstrated that exposition to these NPs caused membrane damage, morphological changes, gene toxicity due to the formation of ROS and alterations in energy-obtaining routes inside cells. However, the appearance of resistance genes to reduce this toxicity was observed, which suggests that, in lower NPs dose, the toxic effect could be tolerated and balanced by the organism. Lin et al. [80] performed a comparative study of toxicity between five different sizes of TiO_2_ NPs (10–50 nm) and crystal phases in *Escherichia coli*, a very ubiquitous bacteria. Results showed that the toxicity was highly dependent on these particle properties, being the anatase form of crystals the one that had the most pronounced toxic effects in *E. coli* from sewage water. The harmfulness of these nanomaterials was dependent on ionic strength and pH of the medium. Finally, this study showed that the damage affected the bacterial cell wall by lipid peroxidation. Similarly, superparamagnetic NPs have been also evaluated. Thus, for example, Frenk et al. [81] assessed CuO and Fe_3_O_4_ NPs toxicity in two different sand soils, using 0.1% and 1.0% concentrations and different nanosystem sizes (<50 nm). Results obtained in both soils differed from each other, but CuO always showed higher toxicity than Fe_3_O_4_. An important reduction of the bacteria hydrolytic activity and oxidative potential was observed, and some bacteria like *Rhizobiales* and *Sphingobacteriaceae* experienced a decrease in their population. On the other side, researchers concluded that organic matter in these soils interacts with NPs, transforming them into lesser toxic agents.

Pure metallic NPs have been also tested. Indeed, Lopes et al. [82] studied the toxic of Au NPs in *Vibrio fischeri* after a 30 min long exposure to concentrations up to 1.67 mg/L. Results showed that growth inhibition depended on exposure time, reporting EC_50_ values (effective concentration were 50% of the inhibition activity is observed) of 0.56, 0.32 and 0.22 mg/L after exposures of 5, 15 and 30 min respectively, concluding that these NPs should be added to the “extremely toxic” grade according to European Union classification. However, Ag-containing nanomaterials have been wider investigated, due to the well-known antibacterial properties of Ag. So, Schlich et al. [83] carried out a study using several Ag NPs concentrations (0.56, 1.67, 5.0 and 15.0 mg/mL) in five different kinds of soils, indicating that the toxicity depends on soil parameters, showing a higher toxic effect in acid, low clay and high sand content. However, researchers were not able to find a direct connection between toxicity and organic compounds in these soils. Beddow et al. [84] demonstrated that Ag NPs produced a reduction in the nitrification potential of *Nitrosomonas europea*, *Nitrosospira multiformis* and *Nitrosococcus oceani*, three ammonia-oxidizing bacterial species. They also discovered that this reduction was concentration-dependent. In another study, Duong et al. [85] analysed the potentially toxic effects of Ag NPs in the cyanobacterium *Microcytis aeroginosa*. Results of SEM-EDX and TEM analysis showed significant morphological changes after exposure to increasing concentration (0.001–1 mg/L) of the Ag-derived nanomaterial. They demonstrated that NPs caused cell lysis and disruption of the cellular membrane, being suitable for removing bloom-forming cyanobacteria from freshwater. The toxic effects of Ag NPs and their transport through the food chain were investigated by Luo et al. [86] using *E. coli* and the nematode *C. elegans* as models. They deduced that the toxicity of Ag particles was dependent on their size: 25 nm in size Ag NPs showed higher penetration and accumulation in *E. coli* compared to 75 nm Ag NPs. In addition, consumption of NP-treated *E. coli* by *C. elegans* resulted in genotoxicity, negative effects in reproduction and toxic effect transferrable to their descendants. Using a little controlled ecosystem, Evariste et al. [87] studied the response that several aquatic microorganisms presented when graphene oxide (GO) NPs in 0.05 and 0.1 mg/L concentrations were introduced in the medium. It was observed that the toxic effect was more pronounced in biofilms rather than isolated bacteria, due to a higher bioavailability in the bacterial community. For example, phylum Planctomycetes growth raised, meaning that these organisms tolerate this nanosystem. On the other hand, researchers found that a higher contamination level caused higher diversity in biofilms because of the different responses that microorganisms showed towards toxicity.

Although to a lesser extent, and due to the proven low toxicity of polymeric materials, the application of polymeric NPs has been studied too. For example, Casado et al. [88] demonstrated that *Vibrio fischeri* did not suffer relevant toxic effects from silicon NPs samples (described as little or non-toxic in the bibliography) nor from polyethyleneimine polystyrene NPs (PS-PEI NPs) samples (described as toxic for several organisms in the bibliography). In similar research work, Naha et al. [89] tested the toxic effects of poly(*N*-isopropylacrylamide) (PNIPAM) and *N*-isopropylacrylamide/*N*-*tert*-butylacrylamide (NIPAM/BAM) polymeric NPs in different proportions, using *Vibrio fischeri* as a model. Although these materials have shown excellent properties as delivery systems for biomedical purposes, the results of the tests carried out by the authors in these bacteria lead to an important toxic effect after a short time of exposure (5 min EC_50_ 40.5 mg/L for 65:35 NIPAM/BAM proportion and 5 min EC_50_ 25.7 mg/L for 50:50 NIPAM/BAM proportion). According to results, to achieve a general conclusion about NPs toxic effects in microorganisms is not an easy task at all, since nanomaterials inherent toxicity substantially differs from each other, between organisms and experimental conditions. In addition, NPs’ intrinsic characteristics such as size, shape, solubility, or surface charge, can suffer modification induced by different conditions, for example, particle agglomeration in presence of an aqueous medium. The presence of all these variables highlights the necessity of rigorous and standardized toxicity evaluation protocols.

### 3.4. Toxicity in Soil Plants and Seaweeds Studies

Plants are a very important group of living beings when it comes to studying any source of environmental impact, due to their interaction with every environmental compartment that can contain NPs such as soil, water, and air. Furthermore, they are an essential part of the food chain, and play an important role in transporting NPs to animals and humans. The most common mechanisms plants use to absorb NPs are through leaves, flowers surface, roots or plant damaged areas. The majority of plant toxicity studies that usually are for human consumption such as corn, wheat or soy are focused on parameter analysis like the rate of seed germination, root growth or nitrogen fixation [53]. Studies show that the concentrations of NPs in the soil are more elevated than in water or air. Therefore, plants are the primary source of NPs to other trophic levels in the food chain [90]. In this sense, Xia et al. [91] tested the impact of three different sized TiO_2_ NPs (21, 60 and 400 nm) in samples of *Nitzschia Closterium* exposed for a period of 96 h. Tested concentrations for 21 nm TiO_2_ NPs ranged from 0 to 100 mg/L, while for the 60 nm size the concentration ranged from 0 to 360 mg/L. In the case of 400 nm BPS NPs, the studies of concentration were in the range of 0–500 mg/L. The EC50 value for the 21 nm NPs is 80 mg/L, for the 60 nm is 125 mg/L and for the 400 nm NPs is 180 mg/L. Results showed an increasing tendency in EC_50_ values, evidencing a reduction in inherent toxicity when NP size increased. Interestingly, TiO_2_ NPs have been evaluated in *Allium cepa* with the following assay focused on possible genotoxicity. Ghosh et al. [92] determined the toxicity after an exposition to different concentrations (0, 2, 4, 6, 8 and 10 mM) to observe that TiO_2_ presented a higher impact in lower concentrations due to NPs precipitation. Further damage produced DNA folding, chromosomal aberrations and ROS production.

Two independent studies concerning ZnO NPs were performed. The first one, dealing with three diatom species exposed for a period of 72 h to four types of ZnO NPs (different sizes and shapes) in concentrations of 10, 20, 40, 80 mg/L to evaluate the rate of growth [93]. The effects on *T. pseudonana* and *C. gracilis* showed growth inhibition, while *P. tricornutum* showed that its growth got reduced proportionally depending on the concentration increase. Wang et al. [94] performed an evaluation where seeded *Arabidopsis thaliana* species in soil were irrigated with ZnO NPs at different concentrations (0–300 mg/L) for 6 weeks. Results indicated that notorious oxidative stress was generated by Zn^2+^ ions in the plant cells, resulting in cellular toxicity. Moreover, all plants showed a NPs concentration-dependent reduction of gene expression, poor level of photosynthesis and retarded growth.

Interesting are the inquiries considering Ag NPs, such as the assays carried out by Turner et al. [95] with *Ulva lactuca.* They demonstrated that, after 48 h exposure, only NPs with higher concentrations (55 mg/L) were able to reduce photosynthetic performance. On the other hand, AgNO_3_ NPs assays showed toxic effects in lower than 2.5 mg/L concentrations, evidencing that the main mechanism of silver-containing NPs is activated when silver is in solution. Other investigations involving Ag NPs in *Arabidopsis thaliana* were performed by Yang et al. [90] who described that although silver-derived nanomaterials negatively affected root elongation but without affect seed germination. Its toxicity mechanism could be explained by the absorption of NPs through the root surface, affecting thylakoid membrane and finally reducing chloroplast content as well as inducing growth inhibition. An additional evaluation was accomplished in wetland plants with a comparative toxicity study of soluble silver (AgNO_3_) versus Ag NPs performed by Yin et al. [96]. They could highlight that arabic gum (6 nm) and polyvinylpyrrolidine (20 nm) coated Ag NPs (40 mg/L) notably affected seed germination and growth of plants. On the other hand, the toxic effect of AgNO_3_ was little or null. The study determined an interesting correlation between the size of the NPs and their toxic effect, due to the ability of smaller sized NPs in crossing plant cell walls. They could conclude that arabic gum coated Ag NPs (6 nm) showed the highest degree of growth retardation of seeds and in soil culture. Yang et al. [97] studied the grown wheat (*Triticum aestivum L.*) with different concentrations of Ag NPs (20, 200 and 2000 mg kg^−1^) in the soil for 4 months. Results showed dose-dependent toxicity, exhibiting severe phytotoxicity with lower biomass, shorter plant height, lower grain weight and decrease in micronutrient content (Fe, Cu and Zn). Another study showed the impact of Ag NPs on chromosomal aberrations [98].

Other NPs as carbon nanotubes have been investigated by the group of Verneuil et al. [99] to evaluate two different carbon nanotubes toxicity, double-wall carbon nanotubes (DWCNTs) and multiple wall carbon nanotubes (MWCNTs), in organisms from *Nitzschia palea* specie. Results proved that these nanotubes produced growth inhibition with inhibitory concentrations ranged from 1 mg (~30% growth inhibition) to 50 mg (~85% growth inhibition) after 48 h of direct exposure. In addition, they evaluated how organic matter present in the medium could affect NPs dispersion and its toxicity, concluding that it would enhance its bioavailability leading to a higher toxic effect. Rico et al. [100] reported that MWCNTs are materials that behave to reduce the cellular density of this specie in a dose-dependent rate. Lower concentrations (20 mg/L) produced cellular apoptosis, while higher concentrations (80 mg/L) produced cell membrane disruption and internal cytoplasm release. Cellular suspension assays showed adaptation to this condition, causing cell aggregations next to the NPs, indicating cellular protection mechanisms.

Other published studies are the oxide NPs such as the ones evaluated by Rui et al. [101,102] concerning full life cycle of the impacts of metal NPs including iron oxide (Fe_2_O_3_) and copper oxide (CuO) at 50 and 500 mg/kg. Results showed that all the nanomaterials caused a decrease in the concentration of total amino acids, except for 50 mg/kg TiO_2_ NPs, when peanut grains (*Arachis hypogaea*) were examined. CuO NPs markedly lowered fresh shoot biomass due to a dose-depended accumulation in the grains. Consequently, metal-based NPs could influence crop yield and quality in a dose-responsive manner.

### 3.5. Toxicity Animal Studies

Performing toxicokinetic and biodistribution studies in animal models are essential to correlate the potential effects of NPs in humans. Aspects like cytotoxicity, genotoxicity, tumorigenesis and reproductive toxicity are investigated [103]. However, most trials and studies carried are performed in vitro, due to the difficulties of experimental animal models, which involve monitorization for months or years and ethics problems because of the high number of animals sacrificed. Toxicity assays in animal models are usually based on blood parameters analysis and their alterations, gene expressions or organ modifications [104].

The impact of Cu, Ag and Au NPs in earthworms (*Eisenia fetida*) has been investigated by Unrine et al. [105] to highlight variations in parameters such as growth, mortality, reproduction rate and genetic expression. Different sized Cu NPs were oxidized in soil and then absorbed by the organisms but without an important effect in concentrations lower than 65 mg/kg. Ag NPs presented significantly different results depending on the type of soil used for the assay. Sandy soil samples presented a higher bioavailability, causing a decrease in growth and reproduction rates in a concentration of 7.4 mg/kg. Nevertheless, in artificial soil samples, reproduction was only affected in concentrations higher than 94 mg/kg. Au NPs with a size between 22–55 nm were accumulated in the organism’s stomach and affected reproduction processes at higher concentrations (50 mg Au/kg soil) after several weeks of exposition.

Aquatic animals such as *Daphnia magna* were exposed to fullerenes and carbon nanotubes, causing intestinal toxicity and movement difficulties, reducing their capacity to swim. This exposure was also related to a loss of functionality in gill tissues, leading to a decrease of the absorbed oxygen and an increase in ROS production [106]. On the other hand, Fraser et al. [107] demonstrated that fish exposed to high carbon nanotubes concentrations in food (500 mg/kg) did not present tissue damage, indicating interestingly that carbon nanotubes toxic effects appeared as a result of the interactions between nanomaterials and organism surfaces. The impact of NPs in crustacean was further tested to check the toxicity of ZnO NPs (26 nm), in concentrations between 4–40 mg/L, after a 96 h exposure in *Tigriopus japonicus* and *Elasmopus rapax larvae* [108]. Toxicity in copepod larvae (*Tigriopus japonicus*) was found to be higher than in amphipod larvae (*Elasmopus rapax*), with CL_50_ values of 0.37 mg/L and 1.19 mg/L, respectively. These results were in concordance with similar cases described in the literature, as most parts of marine organism species are mainly affected by NPs in early growth stages [109]. On the other hand, even though ZnO nanomaterials caused toxicity by themselves, it was demonstrated that Zn^2+^ release was responsible for the most toxicity, which explains why there are some important differences in toxicity with different pH values. Additionally, an in vitro assay was carried out by Canesi et al. [110] in order to determine the effect of polystyrene NPs (50 nm) at different concentrations (1–50 μg/mL) in *Mytilus galloprovincialis* Mediterranean mussel hemocytes. The exposure to these NPs produced intracellular ROS and apoptosis after one hour. The same NPs produced toxic effects in gametes and embryos of *Crassostrea giga* in concentrations between 0.1–25 μg/mL.

Recently, Lehner et al. [111] studied the toxic effects of these polystyrene NPs (70 nm) in zebrafish (*Danio rerio*), in concentrations between 0.025–0.2 μg/mL, causing liver inflammation and fatty acids accumulation. Saddick et al. [112] investigated the effect of the exposure to ZnO NPs of *Oreochromis niloticus* and *Tilapia zillii* fishes. Acute toxicity study with a dose of 14 mg/L caused 100% mortality for both species. Gene expression profiling and analysis of antioxidant property indicated that ZnO NPs at lower concentrations (0.5 mg/L^−1^) showed a significant level of antioxidant properties. When the concentration increased (2 mg/L^−1^), they stopped to express these antioxidant properties. when ZnO NPs were internalized, they caused ROS generation and peroxidation of lipids in brain cells. Furthermore, migratory fishes are one of the most affected organisms by toxicity due to the physiologic changes involved in moving from saltwater to sweet water, in which NPs can affect their digestive and respiratory systems. In order to determine Ag and CuO NPs’ toxicity in migratory fishes, Salari Joo et al. [113] carried out an assay where *Oncorhynchus mykiss* fishes were exposed to these nanomaterials. Results showed a 10% rate of mortality after 10 days of treatment with these NPs at concentrations of 20 mg/L, four times the limit established (4.8 mg/L). Ag NPs (17 nm) in different saline concentrations after 14 days showed a higher absorption when saline concentration was raised, being accumulated mostly in liver, gills or kidneys, where ROS were produced, while higher concentrations (100 mg/L) trigger the fishes died. Other authors indicated that the life-stage dependent zebrafish ZnO NPs toxicity [114]. Wang et al. [115] reported that chronic exposure of zebrafish (*Danio rerio*) to 0.1 mg/L of TiO_2_ NPs in the water had a negative impact on their reproductive stages, exhibiting that these nanomaterials caused a reduction in egg production and elevated embryo mortality after 13 weeks. Ates et al. [116] observed that while there were no signs of neurotoxicity in the goldfish species treated with TiO_2_ NPs, they were significantly accumulated in the intestine and gills. In addition, they found that a higher concentration of TiO_2_-derived nanomaterials caused lipid peroxidation and growth retardation. Finally, Jovanovic et al. [117] evaluated the toxicity of injections of TiO_2_ NPs (<25 nm) into *Pimephales promelas* fish at low (2 ng/g) and high (10 μg/g) doses. Results showed that fish treated with this nanomaterial had more susceptibility to suffering infections and finally death. Histopathological and immunological tests revealed that TiO_2_ NPs notably accumulated in the vital organs and affected the immune system. However, important growth inhibition was observed in concentrations higher than 10 mg/L. Other studies carried out in amphibians with carbon nanotubes tested genotoxicity in a *Xenopus laevis* model. Mottier et al. [118] showed that, although oxidative stress and DNA damage was generated after a short period of exposure, there were not important damages nor irreversible changes.

Interesting investigations concerning Ag NPs in Wistar rats were carried out by Thakur et al. [119] including a 90 days study where Ag NPs (5–20 nm) was administered orally to male Wistar rats at a dose of 20 μg/kg. The aim of the study was to determine whether Ag NPs were suitable for oral therapeutic drugs for long-term treatments. Results showed the accumulation of NPs in the lysosomes of sertoli cells. the analysis of rat testicular cells indicated a degeneration of both cytoplasmic and nuclear components, resulting in apoptosis of germ cells. On the other hand, superparamagnetic iron oxide NPs (Fe_3_O_4_ NPs, SPIONPs) and their toxicity in mice species were investigated [104]. After exposure to these NPs coated with dextran, a resonance analysis to determine physiologic changes in these organisms was conducted. It was demonstrated that NPs affected lipids, glucose and aminoacid metabolic routes, causing disruption in kidneys, liver and heart activities. An increase in fatty acids and plasma glucose was observed, while unsaturated fatty acids and triacyl glycerides decreased. Exposure to lower concentrations of SPIONPs produced minor effects such as nausea, vomits or flatulence. Likewise, Di Bona et al. [120] showed how easily NPs can break through the placenta and cause fetus death by SPIONPs accumulation in the liver. A mouse model was used by Chen et al. [121] in order to determine the potential toxicity of three nanoparticle-based T1 MRI contrast agents: gadopentetate dimeglumine injection (GDI), a clinically used gadolinium-based contrast agent (GBCA), and oxide nanoparticle-based contrast agents, extremely small-sized iron oxide nanoparticles (ESIONs) and manganese oxide nanoparticles (MnO NPs). Results showed that clinically used GBCAs induced accumulation of gadolinium in the spleen, liver and kidneys, which could lead to chronic disease, while ESIONs and MnO NPs exhibited a more stable safety profile.

Regarding human cell toxicity, numerous studies have been performed in the last years. Helal-Neto et al. [122] studied the toxicity produced by the exposure of human melanocyte (NGM), fibroblast (FGH) and endothelial (HUVEC) line cells to an acute dose (20 μg/mL) of polylactic acid polymeric NPs or MMSN (polymeric and magnetic mesoporous silica) NPs. In this case, results did not evidence any relevant toxic effects on neither tumor cells nor non-tumor cells. In metallic NPs, Steckiewicz et al. [123] studied Au NPs shape-related toxicity in hFOB 1.19 (fetal osteoblastic cells), 143B and MG-63 (osteosarcoma) cell lines. Au nanostars were the most cytotoxic, whereas Au nanospheres were the less cytotoxic. Meanwhile, Gea et al. [124] studied the toxicity of TiO_2_ NPs in BEAS-2B (human lung epithelial cells), showing toxicity between 20 to 80 μg/mL depending on their characteristics. Furthermore, the studies of Labrador-Rached et al. [125] about Pt NPs toxic effects in HepG2 (human hepatocellular cells) evidenced that these NPs induced a dose-dependent ROS production, with a substantial response associated with the 25 μg/mL condition.

### 3.6. Green Nanotechnology Studies

Green nanotechnology is known as a respectful-with-the-environment group of techniques and methods used to synthesize NPs. In traditional nanosystem production, there are often involved processes that require the employment of toxic agents, such as sodium borohydride, in addition to high pressure and temperature conditions [126,127,128,129], also requiring high amounts of energy. Green synthesis emerges as an alternative to these classical methods using natural species and compounds instead of chemical agents. Among the new proposed production routes, there is a special and growing interest in those that make use of organisms, like bacteria, fungi, and vegetable extracts (109).

The employment of microorganisms as an alternative way to produce and assemble NPs and nanostructures has been studied in the last years. In particular, they synthesize extracellular enzymes, which possess the ability to produce relatively pure NPs with low toxicity and high biodegradability. This fact sets a reliable alternative route to traditional synthesis [130]. Among all the organisms that can be used for NP biosynthesis, some bacteria, fungi, and yeasts highlight because they are able to expel biomolecules with functional groups where metals can be attached. Microorganism’s advantages against other biological methods are based on their easy handling, high growth rate, low cost and environmental toxicity. Fungi are one of the most used microorganisms for this purpose, due to their ability to secrete higher amounts of proteins [131]. Some disadvantages are a consequence of easily culture contamination, the need for larger processes and a lower control in NPs size [132]. On the other hand, the main disadvantage related to green synthetic methods based on bacteria is the limited spectrum of sizes and shapes obtained [133]. In this sense, some *Actinobacter sp.* organisms can synthesize FeO NPs in aerobic conditions, through the synthesis of the enzyme ion reductase, which converts Fe^3+^ to Fe^2+^. By modifying some precursor molecules, their ability to produce Fe_2_O_3_ and Fe_3_S_4_ has also been studied. The yeast *Saccharomyces cerevisiae* has been studied as an organism capable of synthesizing vanadium pentoxide by the interaction produced between ions (V_2_O_7_)^−4^ and functional groups of its cell wall [130]. *Fusarium oxysporum* fungi have been used to produce different sized magnetic NPs at room temperature by the employment of excreted extracellular proteins [134]. Prabhu and Poulose [135] first reported the synthesis of Ag NPs by *Pseudomonas stutzeri* AG259 strain.

Green synthesis of Ag NPs has been widely studied. For example, Otari et al. [136] investigated the synthesis of Ag NPs using *Rhodococcus spp.* strains. They developed the process at room temperature after 10 h of incubation and obtained spherical nanosystems with a diameter between 10–12 nm. Wang et al. succeeded in the synthesis of Ag NPs in the culture supernatant of *Bacillus methylotrophicus* in 48 h. Li et al. [137] synthesized Ag NPs in the culture of *Aspergillus terreus*. An extracellular enzyme mediated the synthesis, and their diameter was 1–10 nm. Kumar et al. [138] developed a green synthesis technique using the fungus *Phomopsis liquidambaris*. As a result, they obtained spherical Ag NPs with an average size of 18.7 nm. Other research work with algae species, such as *Chaetoceros calcitrans* and *Tetraselmis gracilis*, and their ability to synthesize silver NPs have been reported [132]. In addition, a toxicity comparative study between biosynthesized and chemical synthesized Ag NPs was carried out by Rajasekharreddy et al. [139] Biosynthethic Ag NPs were obtained using *Piper betle* L. leaf extract and their toxicity to *Daphnia magna* was tested. The authors concluded that the presence of protein shells in the Ag core of these nanoparticles is responsible for decreasing the toxic effect that they present in comparison with chemically synthesized ones.

### 3.7. Vegetable Extracts

The ability to produce NPs in vegetable extracts is provided by the reducing agents naturally existing in seeds, fruits and leaves composition. The employment of these compounds leads to NPs with a higher stability level than those produced by microorganisms. Usually, vegetable extracts are preferred for high-scale synthesizing processes because the reducing agent is more concentrated in plant extracts [140]. In this sense, several studies have reported a metallic Fe NPs synthesis produced by a polyphenol extracted from *Camellia*, a very affordable resource that reduces the environmental impact. Interestingly, without the use of surfactants or polymer molecules, NPs were obtained at room temperature due to the effect of reducing polyphenols that also acted as aggregation inhibitors [134]. *Stevia rebaudiana* aqueous extract has been used to prepare porous metallic oxides. Thus, steviol glycosides act like biologic templates because of their hydrophobic and hydrophilic chain fragments, which can interact with inorganic species producing nanorods or nanofibers. Some other examples of vegetable extracts used in NPs production are *Malus domestica*, *Vitis vinifera* and *Solanium nigrum*, whose extracts have been used to synthesize successfully iron NPs [132]. Recently, an ecological and interesting biosynthesis of stable FeNPs, ranging between 2–30 nm, derived from *Phoenix dactylifera* L. extract has been published by Prof. Guerrero’s Laboratory, acting as important reducing and capping agents bearing significant antioxidant activity [141].

## 4. Environmental Positive Impact

Although NPs´ possible negative impact on the environment is relevant, they also can have a beneficial impact that should be thoroughly considered. NPs can have a positive influence on their surroundings with some of their applications. Among the wide range of potential activities NPs can possess to reduce pollution and enhance environmental health, such as promoting production systems with less energy expenditure or helping in the process of water and soil remediation, we have focused on those activities with high relevance and interest in actual research.

### 4.1. Soil and Water Contamination

NPs can be employed in several ways aiming to reduce soil pollution, like remediation processes to diminish the employment of pollutant products [142]. Applying nanotechnology to fertilizers, herbicides, pesticides or growth promoters can lead to important benefits by reducing the required amount of these compounds and enhancing their activity [143]. In this way, the dose needed to achieve the effect is lowered as well as the consequent side effects and pollutant release to the environment and, therefore, the energy that would be consumed to carry out later soil treatments. Some examples are Zn and Al NPs used as coating agents to produce a controlled release of nutrients and the employment of mesoporous silica to protect the pesticides active components against photodegradation [144].

Soil remediation has become one of the NPs most important applications to stop environmental damage. Some nanomaterials that are useful in this remediation process are Zn NP, possessing the ability to degrade dyes and drugs in an effective way [131]. Iron NPs for removing heavy metal or organochlorine compounds by electronic donation has also been studied [145]. In this line, FeO NPs have proved to be effective in Cr(VI) adsorption, with a 98.1% efficiency [134]. Many of these nanomaterials are also biodegradable, not releasing toxic residues [144]. As for soils, the nano-remediation tool possesses a high potential in water decontamination by reducing costs and time expended and can be applied in situ. This in situ remediation entails the direct employment of nanomaterials in water to transform a detoxify pollutant agents [146]. Carbon nanotubes have been studied for this purpose. Their high surface-volume ratio due to their porousness grants a high adsorption capacity, which allows us to eliminate organic and inorganic compounds and makes them ideal candidates for nano-filters [147]. Another example is the use of graphene oxide NPs. This nanosystem allows the adsorption of many organic pollutants such as polycyclic aromatic hydrocarbons, gasoline or dyes [148], in addition to heavy metals, nitrogenous compounds or pesticides [145]. Iron has also been tested. Metallic iron has proved to be one of the most interesting materials for decontamination and water treatment, being useful for organochlorine compounds, arsenic or petroleum derivates [134].

### 4.2. Energetical Applications

Nowadays, the high demand for energy stimulates researchers to find new ways to obtain energy by an environmental-respectful method. In this sense, nanotechnology has become an opportunity to develop clean and renewable energies. Solar photovoltaic energy collected from sun radiation has become the most interesting energy source due to its efficiency, versatility and easy implementation without important impact on natural resources such as water or soils [149]. Some of the major problems that come along with photovoltaic cells involve their high production costs and their low solar energy absorption efficiency, with only 40% becoming electric energy [150]. Carbon nanotubes have been studied as an alternative material for cell structuring since they are easily synthetized, and they can efficiently obtain and stock energy. Furthermore, they withstand temperature changes and sudden shocks [147]. There is also a growing interest in TiO_2_ NPs, due to their excellent adsorption and good photocatalytic activity, making them a potential material for collecting solar energy [151]. On the other hand, carbon nanotube employment in windmills has reported several advantages, such as light-weighted blades that can be enlarged to obtain collected energy or as base products for antifreeze paints that make windmills to last for a longer period of time in cold climates. Geothermal energy obtainment is also favored with the use of some NPs that decrease the depth and temperature needed to collect this energy [150].

### 4.3. Other Applications

Sensors present in the different environmental compartments are a very powerful tool to detect contaminants [152] such as pesticides [153], antibiotics or microorganisms [154]. The employment of nanomaterials for this purpose enhances some characteristics of these sensors, such as sensibility or precision, due to their high surface and reactivity [155]. Even though this technology is still in its very early stages, some studies conclude that the implementation of metallic NPs (such as gold, silver or cobalt NPs) could lead to a considerable improvement in actual sensors [156].

On the other hand, despite the increase of clean and renewable energy sources available, the greatest part of the industry still uses fossil fuels, which obliges to minimize the damage they generate by collecting and stocking part of the CO_2_ emitted to the atmosphere. Nanotechnology emerges as a different approach to this problem, and several solutions have been studied, such as the employment of high permeability and selectivity polymeric membranes with nano-holes or the use of ZIF (Zeloitic Imidazolare Frameworks) NPs [149].

## 5. Legislation

Nanotechnologies involve both benefits and risks. However, whether benefits have been widely studied by many researchers, risks are not well known. Taking into account the risk-benefit balance, it is necessary an evaluation to determine if the employment of NPs is justified or, on the other hand, it should be strictly regulated due to their potential impact in the environment and human health. There is an urgent need for balance to fit between both. A very strict regulation could prevent further developments and technologies, and a very soft regulation could lead to massive environmental pollution.

NPs regulation is carried out by several organizations, depending on how and where they are employed or fabricated. For example, in the USA, Food and Drugs Administration (FDA) [157] is responsible for regulating NPs employment in food, medicines and cosmetics. On the other hand, the Environmental Protection Agency of the United States [158] takes over the emissions in the air, water and soil, and the waste produced by chemical products in the farming industry. This regulation faces several difficulties, such as different NPs types that impede the development of general rules and laws, controlling every environmental emission pathway including regular employment attrition and lack of consolidated information about environmental impact and long-term risks [159].

In 2011, an analysis of European legislation [160] concluded that the regulations in force are not specific enough, possibly due to this lack of information. This study also highlighted the difficulties involved when trying to monitor the existence of NPs in the various environmental compartments. In this line, the need of facing these problems in the legislation, on one hand, by researches in order to obtain reliable data and, on the other hand, by establishing policies that take into account NPs particular characteristics and the risks they involved.

Aiming to this objective, initiatives like Nanodatabase [161] are emerging with the economic help of organizations like the European Research Council and the Danish Consumer Council. Nanodatabase classifies products depending on their impact in the environment and in human health. By using a color code (red, yellow, and green) they inform about both their emission and potential toxicity. In 2019, they classified 3377, rising more than 3400 in the four first months of 2020.

## 6. Conclusions

NPs emission to the environment can occur anytime during their life cycle, but the impact is harder to determine when the products are employed by the consumer. Transformations play an important role in NPs physicochemical properties, resulting in an environmental impact that could differ from which original NPs may cause. Because of that, they should be thoroughly taken into consideration the toxicity studies. On the other hand, although toxicity mechanisms depend on NPs nature, most of them converge in oxidative stress mechanisms, leading to ROS formation and damaging cell structures. There are plenty of in vivo and in vitro studies that support the evidence of NPs’ potential toxicity in a wide variety of living beings, affecting cell structure in different ways. Effects caused by NPs toxicity differ from each other, not only depending on the nanomaterial characteristics, but also on the target organisms. The way that organisms suffer this impact and the response induced, which greatly depends on how they are exposed to the toxic agents, makes it difficult to generalize the environmental impact of NPs emissions. In this way, research has shown that the most effective way of evaluating this impact undergo an individually focused evaluation of each pollution case. Nevertheless, some NPs exhibit characteristics that impact the environment positively depending on their usages, leading to a reduction of pollution, energy waste and the greenhouse effect.

Finally, it is important to highlight that the actual legislation about these pollutants is obsolete. Although the development of new and precise laws that strictly regulate NPs emission is highly necessary, is essential maintaining a balance between sustainable development and scientific progress

## Data Availability

Data sharing is not applicable to this article.
