# Erratum: Guillermo M., et al. Environmental Impact of Nanoparticles’ Application as an Emerging Technology: A Review. *Materials* 2021, *14*, 166

**DOI:** 10.3390/ma14071710

**Published:** 2021-03-31

**Authors:** Guillermo Martínez, Manuel Merinero, María Pérez-Aranda, Eva María Pérez-Soriano, Tamara Ortiz, Eduardo Villamor, Belén Begines, Ana Alcudia

**Affiliations:** 1 Department of Organic and Medicinal Chemistry, Faculty of Pharmacy, University of Seville, C/Profesor García González, 2, 41012 Seville, Spain; mtnezmun@gmail.com (G.M.); lolo191995@gmail.com (M.M.); mariapar89@gmail.com (M.P.-A.); 2 Department of Materials Science and Engineering and Transport, Escuela Politécnica Superior, University of Seville, 41011 Seville, Spain; evamps@us.es; 3 Department of Normal and Pathological Cytology and Histology, Faculty of Medicine, University of Seville, 41009 Seville, Spain; tortiz@us.es; 4 Faculty of Pharmacy, University of Seville, C/Profesor García González, 2, 41012 Seville, Spain; eduardovillamors@gmail.com

The authors wish to make the following corrections to this paper [1]:

Change in Author Names (Add a new one)

In the original version of our article [1], the student Eduardo Villamor was not included as an author. To correct this oversight, Eduardo Villamor has now been added to the authorship of the manuscript. The acknowledgements and authors contributions have also been modified. 

The corrected author list, affiliation list, authors contribution, and acknowledgements are provided below:

##  

MartínezGuillermo^1^MerineroManuel^1^Pérez-ArandaMaría^1^Pérez-SorianoEva María^2^OrtizTamara^3^VillamorEduardo^4^BeginesBelén^1^*AlcudiaAna^1^*1Department of Organic and Medicinal Chemistry, Faculty of Pharmacy, University of Seville. C/Profesor García González, 2, 41012 Seville, Spain; mtnezmun@gmail.com (G.M.); lolo191995@gmail.com (M.M.); mariapar89@gmail.com (M.P.-A.)2Department of Materials Science and Engineering and Transport, Escuela Politécnica Superior, University of Seville, 41011 Seville, Spain; evamps@us.es3Department of Normal and Pathological Cytology and Histology, Faculty of Medicine, University of Seville, 41009 Seville, Spain; tortiz@us.es4Faculty of Pharmacy, University of Seville. C/Profesor García González, 2, 41012 Seville, Spain; eduardovillamors@gmail.com*Correspondence: bbegines@us.es (B.B.); aalcudia@us.es (A.A.)

**Authors** **Contributions:** Conceptualization and methodology, B.B. and A.A.; investigation, G.M., M.M., M.P.-A., and E.V.; writing—original draft preparation, G.M., M.M., M.P.-A., E.M.P.-S., T.O., and E.V.; writing—review and editing, T.O. and E.M.P.-S.; supervision, B.B., A.A.; funding acquisition, A.A. All authors have read and agreed to the published version of the manuscript.

**Acknowledgments:**  University of Seville Library for providing access to scientific and technical information resources. Some figures have been designed using resources from Freepik.com.

The authors would like to apologize for any inconvenience caused to the readers by these changes.
